# Patient preferences for breast cancer referral: development and pilot study

**DOI:** 10.1186/1753-6561-9-S7-A26

**Published:** 2015-10-27

**Authors:** Emma Aherne, Kirsty O'Brien, Aisling Walsh, Ronan McDonnell, Rose Galvin, Tom Fahey

**Affiliations:** 1 HRB Centre for Primary Care Research, RCSI, Dublin, Ireland

## Background

Levels of referral to specialist breast clinics in Ireland have increased from 23,575 referrals in 2006 to 37,631 referrals in 2010 [1]. Over this period however, levels of breast cancer diagnosis have remained relatively consistent [1]. This means that more women are being exposed to potentially harmful investigations without an increase in benefit. The aim of the project was to assess patient preferences for a watchful waiting programme versus immediate referral to a specialist breast clinic for triple assessment, if presenting to their General Practitioner with breast symptoms that have a low risk of malignancy.

## Methods

A combination of literature review and expert opinion were used to create the health states and decision tree. We used a combination of a visual analogue scale (VAS), time trade-off analysis (TTO) and standard gamble (SG) to measure utilities. The health states and utility assessments were incorporated into a computer programme designed in house. Patient preferences were assessed using this programme as well as written explanations, verbal discussions and visual aids. Pilot interviews were performed with healthy participants. These interviews were to test the interview style, understanding of health state scenarios, utility assessment and statistical methods.

## Results

A decision tree was developed that involved one decision node and nine choice nodes. There were 11 health states developed that used a standard format with information on diagnosis, investigations and the physical and emotional consequences of the health state. Five pilot interviews were performed. Our main findings were that participants had some difficulty comprehending TTO, but understood the SG better. Participants found difficulties using the laptop administered programme; most participants found some of the questions emotionally difficult, and needed time to discuss any issues that had arisen after the interview was completed.

## Conclusions

A decision tree was designed which incorporated the most relevant outcomes necessary to the research being undertaken. Further research is needed to explore patient preferences around referral from primary to secondary care with breast symptoms. However, this research may serve to identify a more rational approach to referral to specialist breast units where women presenting with low risk symptoms could choose to undergo either a programme of watchful waiting with their GP or immediate referral for triple assessment.
